# Supplementary material for: High Jc and low anisotropy of hydrogen doped NdFeAsO superconducting thin film
Source: Sci Rep. 2021 Mar 11;11:5636. doi: 10.1038/s41598-021-85216-3 (PMC7952916; doi:10.1038/s41598-021-85216-3)
Supplement: Supplementary file 1 — Supplementary Information [file 41598_2021_85216_MOESM1_ESM.pdf]

Supplementary information on "High J_c and low anisotropy of hydrogen doped NdFeAsO superconducting thin film"

Kazumasa Iida[1,6], Jens Hänisch[2], Keisuke Kondo[1], Mingyu Chen[1], Takafumi Hatano[1,6], Chao Wang[3], Hikaru Saito[4,6], Satoshi Hata[3,5,6], and Hiroshi Ikuta[1]

[1]*Department of Materials Physics, Nagoya University, Chikusa-ku, Nagoya 464-8603, Japan*
[2]*Institute for Technical Physics, Karlsruhe Institute of Technology, Hermann-von-Helmholtz-Platz 1, 76344 Eggenstein-Leopoldshafen, Germany*
[3] *The Ultramicroscopy Research Center, Kyushu University, Nishi-ku, Fukuoka 819-0395, Japan*
[4]*Institute for Materials Chemistry and Engineering, Kyushu University, Kasuga, Fukuoka 816-8580, Japan*
[5]*Faculty of Engineering Sciences, Kyushu University, Kasuga, Fukuoka 816-8580, Japan*
[6]*JST CREST, Kawaguchi, Saitama 332-0012, Japan*

1. The effect of microbridge processing on the superconducting transition temperature.

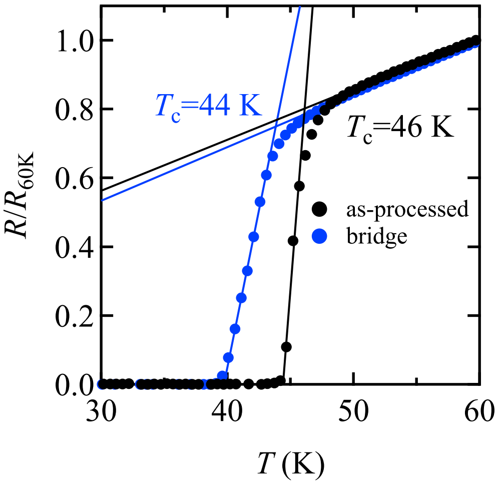

Figure S1. Normalised resistance as a function of temperature. The as-processed sample showed a T_c of 46 K. After fabrication, the bridge sample showed a T_c of 44 K.

2. The pinning force analysis.

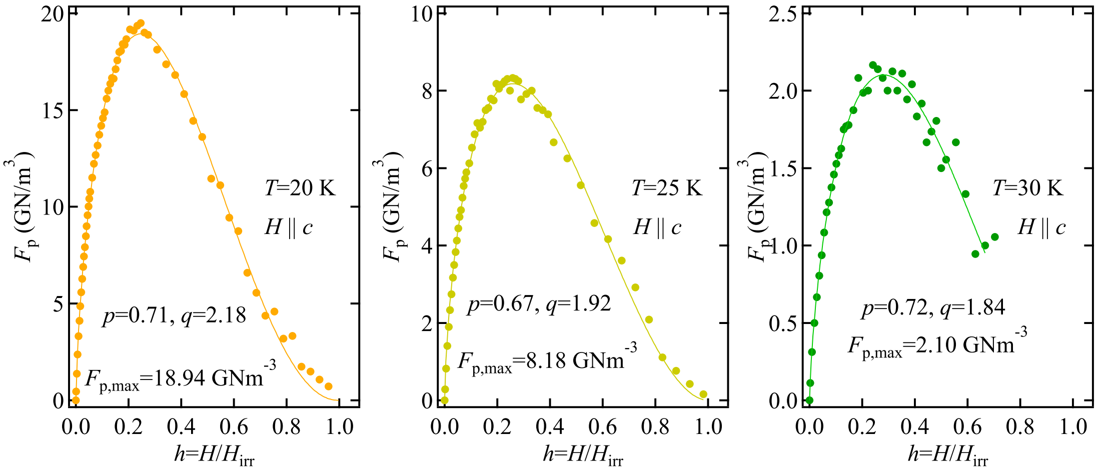

Figure S2. Pinning force density F_p as a function of reduced field for H || c. Exponents p and q in $h^p(1\text{-}h)^q$ are evaluated for each temperature. The results are summarised in fig. 4e.

3. Determining the dimensional cross-over temperature T_{cr}.

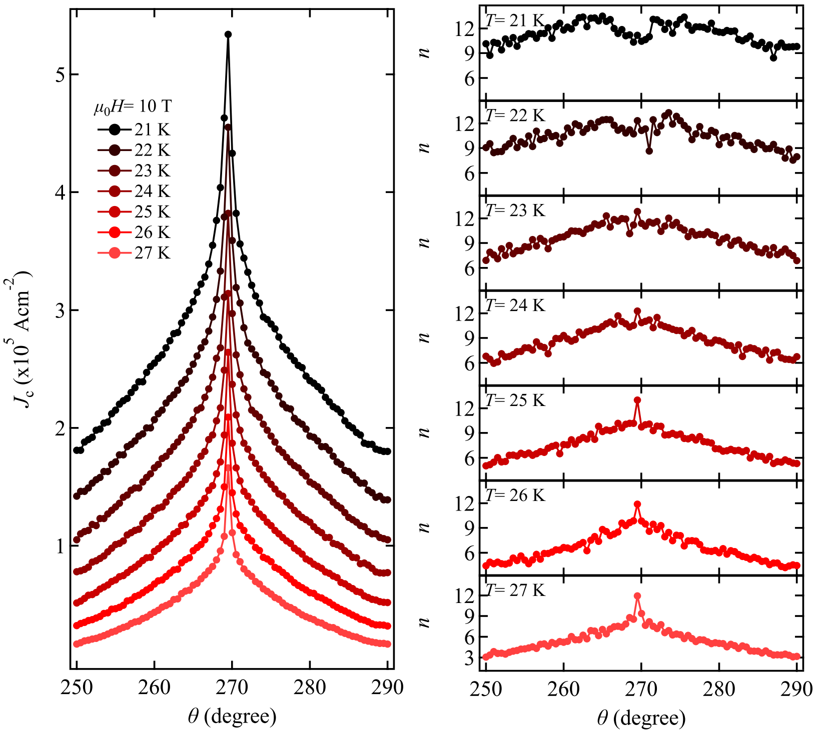

Figure S3. Angular dependence of J_c and the corresponding n under a fixed magnetic field of 10 T measured at various temperatures. As the temperature decreases from 27 K, the exponent n around θ~270° (i.e. H || ab) starts showing shoulders at 24 K, followed by a dip formation.

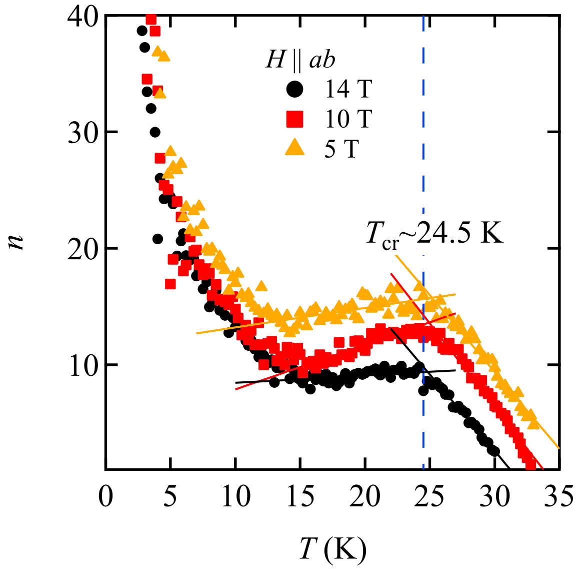

Figure S4. Temperature dependence of *n* for *H* || *ab* under several magnetic fields. The exponent n increases with decreasing T and stays constant around 24.5 K. Below 15 K, n starts to increase again with decreasing T.

4. *E-J* curves for determining J_c.

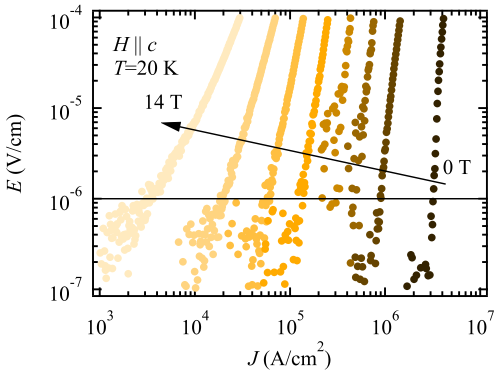

Figure S5. Representative *E-J* curves. *E-J* curves at 20 K for $H \parallel c$. Field increment was 2 T. J_c was determined as the intersection between E=1 μV/cm and each curve.
